# Factors affecting the quality of life of adults living with congenital adrenal hyperplasia: a qualitative study of lived experience

**DOI:** 10.1530/EC-26-0033

**Published:** 2026-05-12

**Authors:** K Lynette James, Nicola Parkin, Sue Elford, Christine McKnight, Rhiannon Phillips, Timothy Pickles, S Faisal Ahmed, Nils Krone, Sofia Llahana, Michael W O’Reilly, Jeremy W Tomlinson, D Aled Rees

**Affiliations:** ^1^ Cardiff and Vale University Health Board, Cardiff, United Kingdom; ^2^ Cardiff University, School of Pharmacy & Pharmaceutical Sciences, Cardiff, United Kingdom; ^3^ Cardiff University, School of Psychology, Cardiff, United Kingdom; ^4^ Living with CAH, CAH Patient Support Group, Cambridge, United Kingdom; ^5^ Cardiff Metropolitan University, Cardiff School of Sport and Health Sciences, Cardiff, United Kingdom; ^6^ Leeds Beckett University, School of Humanities and Social Sciences, Leeds, United Kingdom; ^7^ University of Glasgow, Developmental Endocrinology Research Group, Glasgow, United Kingdom; ^8^ University of Sheffield, Division of Clinical Medicine, Sheffield, United Kingdom; ^9^ City St George’s University of London, School of Health and Medical Sciences, London, United Kingdom; ^10^ Royal College of Surgeons in Ireland (RCSI), Department of Medicine, Dublin, Ireland; ^11^ Oxford Centre for Diabetes, Endocrinology and Metabolism, NIHR Oxford Biomedical Research Centre, University of Oxford, Oxford, United Kingdom; ^12^ Cardiff University, Neuroscience and Mental Health Innovation Institute, Cardiff, United Kingdom

**Keywords:** congenital adrenal hyperplasia, CAH, health-related quality of life, lived experience, patient-reported outcomes

## Abstract

**Objective:**

Congenital adrenal hyperplasia (CAH) is a genetic condition caused by enzymatic defects of adrenal steroidogenesis. The physical manifestations of CAH are well recognised, but the effects on health-related quality of life (HRQoL) are unclear. We sought to explore the factors impacting the HRQoL of individuals with CAH.

**Design:**

Phenomenological qualitative study of lived experience.

**Methods:**

In-depth, timeline-assisted, semi-structured interviews were undertaken virtually with participants recruited via the Living with CAH patient support group. Participants, purposively selected until data saturation, were adults (≥18 years) with CAH and parents or partners of adults with CAH. Interviews were audio-recorded, transcribed verbatim, and analysed using framework analysis.

**Results:**

Twenty-three participants were interviewed (20 classic, 1 non-classic, and 2 mothers). Most participants (*n* = 19) were female. CAH has a profound physical, psychological, and psychosocial impact on individuals. The psychological well-being of women was compromised by trauma from childhood medical examinations and a lack of agency in treatment decisions. Poor self-esteem, shame, and negative body image impaired female social functioning. Female sexual dysfunction from genital malformation or surgery and psychosexual issues negatively impacted intimate relationships. Fertility and reproductive choices were a concern to both sexes. Complex family dynamics with dependent relationships were evident.

**Conclusion:**

This study identified a breadth of factors impacting HRQoL in CAH – domains overlooked by an existing measure that predominantly focuses on physical symptoms. Further work is needed to develop a sensitive, comprehensive disease-specific HRQoL measure that reflects the lived experience of individuals with CAH to facilitate delivery of patient-centred care and improved patient outcomes.

## Introduction

Congenital adrenal hyperplasia (CAH) is a group of rare autosomal recessive disorders caused by enzymatic defects in adrenal steroidogenesis, resulting in impaired cortisol synthesis and androgen excess in many cases (1). The most common form (∼90%) is due to a CYP21A2 mutation causing 21-hydroxylase deficiency, characterised by reduced cortisol, elevated androgens, and, in some individuals, reduced aldosterone concentration (2). Other less common causes include deficiencies in 17α-hydroxylase, 11β-hydroxylase, 3β-HSD, StAR protein, and P450 oxidoreductase (1). Clinical severity varies by enzyme defect and residual activity (3).

CAH is associated with considerable morbidity. Individuals who are life-dependent on glucocorticoids are vulnerable to adrenal crises, particularly during acute illness, trauma, and surgery (2). Glucocorticoid over-replacement can lead to metabolic complications, such as obesity, hypertension, dyslipidaemia, osteoporosis, and insulin resistance (4). Gonadal dysfunction, including hypogonadism, impacts both sexes, contributing to infertility (5). In women, infertility may be compounded by anatomical and psychosexual issues stemming from genital anomalies and feminising surgery. However, fecundity in women with CAH is similar to that of the general population (1). Broader issues impacting female psychosexual function include gender identity, sexual orientation, intimate relationships, pregnancy, and parenthood (6). Psychosocial outcomes are sub-optimal in CAH, with individuals reported to have lower academic attainment, employment, and income, alongside higher sick leave and disability claims (7, 8).

Given the physical, psychosexual, and psychosocial burden, CAH would be expected to significantly impair health-related quality of life (HRQoL). However, research using generic instruments (e.g. SF-36, EQ5DL, and WHO-Bref) is conflicting due to differences in clinical practice, support systems, and methodologies (8, 9, 10, 11, 12, 13, 14, 15, 16, 17, 18, 19, 20, 21, 22). These generic instruments also lack the specificity and sensitivity to effectively capture the diverse symptomatology and psychosocial impact of CAH. A recent CAH-specific HRQoL patient-reported outcome measure (PROM), adapted from instruments for Addison’s and Cushing’s disease, focuses mainly on physical symptoms (23). Psychometrically tested in a cohort of 69 adults, this PROM omits key HRQoL domains relevant to CAH (e.g. psychological trauma, gender dysphoria, fertility, and pregnancy) (23). Consequently, the validity of this measure remains uncertain. To effectively support adults with CAH and improve HRQoL, clinical teams require more specific and holistic PROMs that are grounded in the lived experience of individuals with CAH. This study aimed to provide a comprehensive account of the factors influencing HRQoL by exploring the lived experiences of individuals with CAH.

## Methods

This study is reported in line with the Consolidated criteria for reporting qualitative research (COREQ) standards (24). Ethical approval was provided by Cardiff University School of Medicine Research Ethics Committee (ref number: 24/32).

### Study design

Phenomenology was adopted as the research paradigm because it focuses on understanding the lived experience of a phenomenon from the participant’s perspective (25). An experienced female qualitative researcher (NP) conducted one-to-one, timeline-assisted semi-structured interviews with participants (26, 27). The researcher had no personal experience of CAH, and a reflexive stance was adopted throughout data collection to minimise bias from preconceived ideas.

### Study participants: sampling strategy and eligibility criteria

A purposive sample of adults affected by CAH was recruited via the Living with CAH patient support group (28). The inclusion criteria were adults 18 years of age and over diagnosed with any form of CAH or a parent or a partner of an adult with CAH. Individuals under the age of 18 years were excluded. While a formal sample size is not necessary for qualitative research, recruitment continued until interview data indicated theme saturation was achieved (29).

To recruit participants, the research team provided Living with CAH with an email for distribution to their membership and a poster for display on their website and social media. Both the email and poster included the research team contact details and a hyperlink to the digital participant information leaflet (PIL) and consent form. There were separate PILs and consent forms for (i) participants with CAH and (ii) partner or parent of an adult with CAH. Written informed consent was obtained from participants prior to the interview.

### Development of the interview schedule

The interviews were designed to be naturalistic conversations, where participants felt in control of the conversation and psychologically safe to discuss sensitive experiences. A flexible interview schedule was developed to aid the interviewer navigate sensitive topics. The interview schedule, developed iteratively by [KLJ], was informed by a literature search, informal online discussions with four Living with CAH committee members (involving [KLJ] and [DAR]), and input from psychologists with expertise in sexual and reproductive health ([CMcK], [RP]). The interview schedule prompted exploration of the impact of CAH and its treatment on the following topics: physical, emotional, and mental health; gender identification, sexuality and intimate partner relationships; reproductive choices; and sources of support, including family and social relationships.

### Data collection procedure

**Figure 1:**
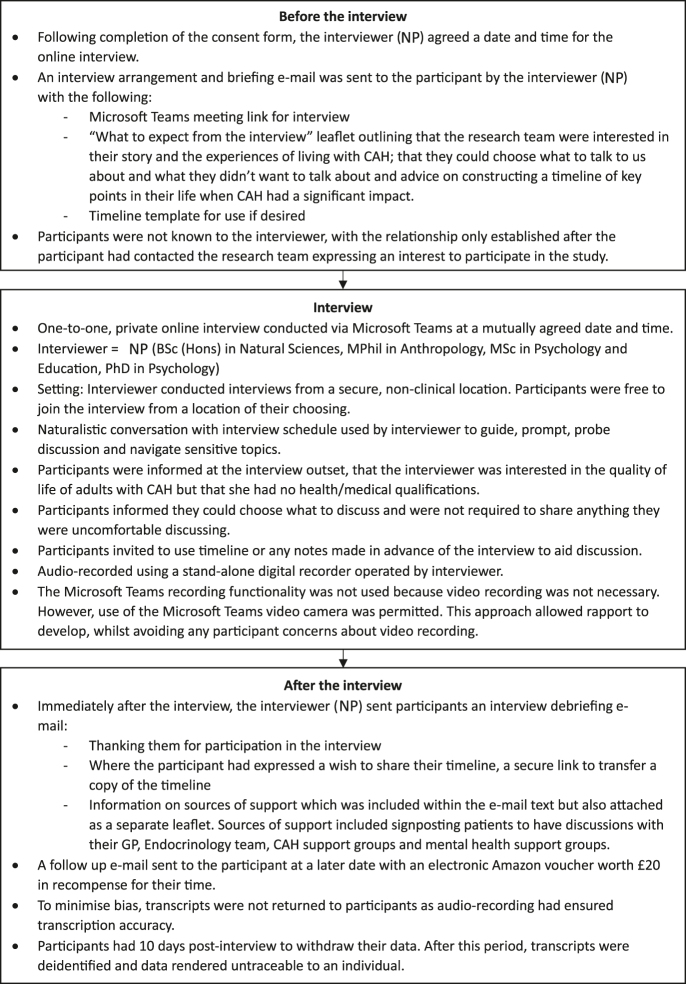
Stages of the data collection procedure. A flow diagram outlining the different stages of the data collection procedure, including before the interview, during the interview, and after the interview.

Figure 1 outlines the data collection procedure. Interviews were conducted online (October–December 2024) and audio-recorded using a standalone digital recorder. The interview was opened by inviting participants to ‘Tell me your story’. Before the interview, participants were encouraged to prepare a timeline of significant life events impacted by CAH. This promoted participant agency over the discussion and exploration of changes across the life course. During the participant’s oral history, the interviewer remained minimally intrusive, using prompts from the interview schedule to guide the conversation and noting observations (e.g. body language and emotional responses) in field notes.

### Data analysis

**Figure 2:**
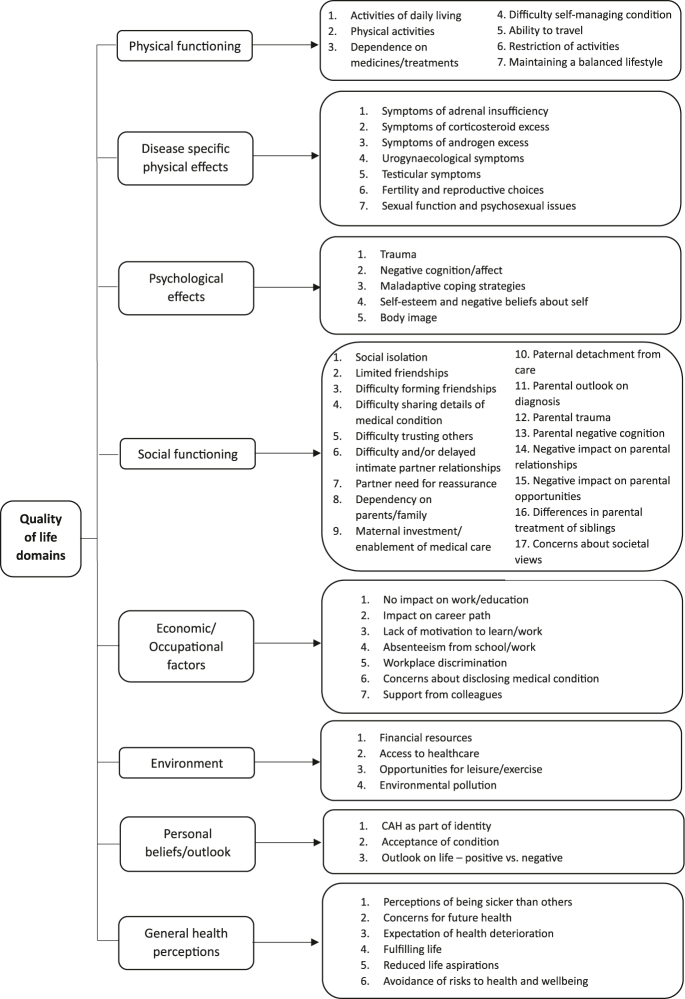
Summary of themes relating to factors affecting quality of life in CAH. The diagram maps the factors impacting quality of life in adults with CAH to health-related quality-of-life domains.

Interview audio-recordings were transcribed verbatim, and relevant field notes were added before deidentification (Fig. 1). The transcripts were entered into NVIVO 14 for analysis. Data were analysed using framework analysis (30, 31). Inductive thematic analysis was undertaken to identify HRQoL themes from participant stories, free from preconceived ideas or theories (32). A thematic framework of factors affecting HRQoL was constructed from the themes identified. This framework was systematically applied to the data with themes refined by indexing, charting, and mapping to HRQoL domains. Dual coding of data was undertaken independently by [NP] and [KLJ], to validate coding accuracy, with differences resolved by discussion. The coding tree consisted of a parent code (HRQoL domain) that encompassed nuanced sub-themes reflecting participant experiences (Fig. 2).

## Results

Thirty-one individuals expressed interest in participating, with 29 meeting the inclusion criteria. Twenty-three participants responded to further communication and were interviewed. Table 1 summarises participant characteristics. Twenty-one participants had CAH (classic = 20; non-classic = 1), and two were mothers of female study participants. Most participants identified as women (*n* = 19). Three women were incorrectly assigned male at birth, with one raised as male until the age of seven. All participants had received healthcare in the UK, although several had experiences of other healthcare systems as residents in the United States of America (USA), Canada, Switzerland, Germany, India, and Japan.

**Table 1:** Participant characteristics.

Participant identifier	Gender	Age	CAH variant	Genital surgery
1	Female	28	Classic – salt wasting	No
2	Female	34	Classic – salt wasting	Yes – childhood and adult
3	Female	61	Classic – salt wasting	Yes – childhood
4	Male	41	Classic – salt wasting	N/A
5	Female	Mid-fifties	Classic – virilising	Yes – adult
6	Female	35	Classic – salt wasting	Yes – childhood
7	Female	37	Classic – virilising	No
8	Female	Twenties	Classic – salt wasting	Yes – childhood and adult
9	Female	39	Classic – virilising	Yes – childhood and adult
10	Female	61	Classic – salt wasting	Yes – childhood
11	Male	25	Classic – virilising	N/A
12	Female	62	Classic – virilising	Yes – childhood
13	Female	57	Classic – virilising	Yes – childhood
14	Female	N/A	Parent	N/A
15	Female	53	Classic – virilising	Yes – childhood
16	Female	60	Classic – salt wasting	Yes – childhood
17	Female	45	Classic – virilising	Yes – childhood
18	Female	Late forties	Classic – salt wasting	No
19	Female	N/A	Parent	N/A
20	Female	29	Classic – virilising	Yes – childhood
21	Male	38	Classic – salt wasting	N/A
22	Male	37	Classic – virilising	N/A
23	Female	Late thirties	Non-classic	N/A

Interviews lasted an average of 50 min (range: 26–71 min), ending when participants had no further issues to discuss. Theme saturation was apparent after 23 interviews. Figure 2 summarises the identified factors affecting HRQoL in CAH.

### Physical functioning

CAH had minimal impact on participants’ ability to perform activities of daily living or physical activities, with some participants partaking in demanding sports (e.g. martial arts, mountain biking, and skydiving). All participants cited dependence on medication/treatment (Table 2). Taking medication, attending hospital appointments, and undergoing medical interventions (e.g. examinations, blood tests) were viewed as the norm. Some participants required additional treatment for glucocorticoid side effects (e.g. osteoporosis and reflux). Medication adherence was challenging for some individuals who reported forgetting to take their medication. However, participants often carried spare medication. If they had insufficient medication in their possession, participants would decline unexpected social invitations that they would otherwise have accepted (Table 2). Few participants (*n* = 2) were concerned about their ability to self-manage the condition, with independent administration of intramuscular hydrocortisone a specific concern. CAH did not affect participants’ ability to travel, who confidently travelled overseas. However, two participants were discouraged from travelling to low-income countries with limited access to healthcare. Maintaining a balanced lifestyle was important to participants. While participants felt that having CAH should not restrict life, two participants limited their use of alcohol due to a fear of vomiting and adrenal crisis (Table 2).

**Table 2:** Summary of key sub-themes relating to the physical functioning and disease-specific physical effect domains.

Health-related quality-of-life domain	Sub-theme	Illustrative quote
Physical functioning	Physical activities	*‘I lift weights…I do a lot of exercise… I’ve never gone more than three days, maybe four, without a formal workout…heart pumping!’* (participant 7)
	Dependence on medication and treatment	*‘I’ve known no difference to hospital appointments and physicals…and medication…to me, it’s just the norm…I’ve not known anything different’.* (participant 21) *‘On a night out, my friend would say to me, “Do you want to stay over rather than going back?” and I’d say, “No, I can’t because I haven’t got my medication with me…” so it’s just things like that…’* (participant 1)
	Maintaining a balanced lifestyle	*‘…you just need to try and stay as healthy as you can…without living a life where you feel like you’re constantly restricted…’* (participant 6)
Disease-specific	Fertility and reproductive choices	*‘I’ve never had a period and I won’t have kids because of that… obviously you’ve got surrogacy and adoption…’* (participant 8) *‘I know that having kids can be a bit of a tricky thing… I’m still waiting on the results of fertility’.* (participant 11) *‘The idea of having children…just no... not just getting pregnant, not just the pregnancy, not just the labour…which would all be challenging, with the CAH...but the actual raising of a child is a lifelong thing!..That is so stressful!. And I just feel that I would be sacrificing my health again’.* (participant 23) *‘I feel I’ve got an obligation not to pass it on’.* (participant 22)
	Sexual function and psychosexual effects	*‘It’s painful…the bits of sex I have had… I just have no desire whatsoever to engage in sex’* (participant 3) *‘The only way it held me back a bit is…was with relationships…because I knew I’d had surgery down there a couple of times…I knew that maybe I would look a bit different to other people, so I think I was quite apprehensive about taking things a bit further in relationships…’* (participant 2)
	Gender identity	*‘I was a bit confused about my gender, but these days, I’m like ‘I’m a woman, what the hell? Get through it!’ and as I get older, I feel more strongly about that…but as an early teen’* (participant 13)
	Sexuality	*‘It* [CAH] *had quite an impact on my sexuality…I felt so embarrassed…so it took me time to… to become sexually active…and actually in my earlier years…I kind of was a lesbian…I thought I was a lesbian, and as I got older, I realised…I’m probably pansexual now…I’m married to a man...my sexuality became quite complicated, when I was a teenager and in my twenties…I found it easier to be in the lesbian community... actually as I got older, more confident…I began to explore men as well as women…So I think some of my sexuality was very based on how my body looked rather than all the other connections you have...’* (participant 5)

### Disease-specific physical effects

Participants’ HRQoL was impacted by symptoms of adrenal insufficiency, androgen excess, and iatrogenic glucocorticoid excess (see section on Supplementary materials given at the end of the article). Problematic symptoms of adrenal insufficiency were lethargy, adrenal crises, headaches, hypoglycaemia, and memory issues. Weight gain, osteopenia or osteoporosis, skin concerns (e.g. striae, thin skin, bruising, and acne), immunosuppression, and gastro-oesophageal reflux were commonly reported symptoms of glucocorticoid excess. Metabolic complications of glucocorticoid use (e.g. hypertension and dyslipidaemia) were reported by older participants. Hirsutism, absent or irregular periods, and restricted height were concerning symptoms of androgen excess. Women also reported a variety of urogynaecological symptoms resulting from urogenital anomalies and genital surgery. These included vaginal stenosis, urinary tract infections, incontinence, and labial and vaginal infections. One male referred to testicular adrenal rest tumours (TARTs).

The HRQoL of both sexes was impacted by concerns regarding fertility and reproductive choices (Table 2). Three women reported being unable to conceive because of CAH. In contrast, three women had defied expectations and conceived easily. Some participants had actively decided against parenthood, citing concerns regarding passing CAH to potential offspring and the impact of pregnancy and child-rearing on their health. Other participants were pursuing genetic counselling and pre-implantation genetic screening to avoid passing CAH to their children. Owing to CAH, prospective mothers were aware they would need to deliver in a hospital, and a caesarean section was likely if they had a previous history of genital surgery. Mothers with CAH reported pregnancy complications, including miscarriage, gestational diabetes, and difficult delivery with foetal exposure to glucocorticoids.

Only women reported impairment of sexual function and psychosexual effects (Table 2). Some participants who had undergone genital surgery reported dyspareunia, low libido, anxiety around intimacy, and potential rejection, attributed to perceived genital differences. Seven women reported genital dysmorphia, with one participant undergoing genital surgery in adulthood because she was ‘embarrassed by how everything looks!’ (participant 5). One participant, with no known genital anomaly, questioned, ‘Am I normal?…Was I meant to have surgery?’ (participant 1).

All female participants, including those assigned male at birth, identified with their female anatomical sex (ovaries, uterus, and vagina). Two women noted confusion over their gender identity, particularly as teenagers, because of gender nonconforming tastes in clothes, hobbies, and career. Participants were predominantly heterosexual. However, several women noted challenges understanding their sexuality, with five disclosing non-heterosexual orientation. Participants attributed non-heterosexual orientation to excess androgens and the impact on physical appearances (Table 2).

### Psychological

Trauma from medical examinations, surgery, hospitalisation, and being misassigned the incorrect sex was prevalent (*n* = 18) in women, with avoidance and dissociation common (Table 3). Participants were distressed by their lack of agency and choice in treatment decisions, particularly as children. Consequently, participants reported ‘...a very deep-seated dislike of medics...a deep-seated distrust of doctors’ (participant 13). In contrast, only one man reported feeling embarrassed about having his testicles examined as a teenager.

**Table 3:** Summary of key sub-themes relating to the psychological domain.

Sub-theme	Illustrative quote
Trauma	*‘You’d be asked to take your clothes off…and there’d be a line of doctors…especially students…and they’d all come and have a look, you know…and nowadays that would be seen as pretty abusive, I think…and photographs and things…’* (participant 10) *‘The specialist would look at me…examine me…try and ram a good old dilator in me, which I hated…’* (participant 3) *‘I’ve felt loss of agency, obviously…things are always being done to you…loss of bodily agency…to constantly have adults…strange different adults…doing things to you…you are just like meat that other people mess around with?…it’s abusive…people interfering with you…and you don’t want them to…and you’re being forced to submit to that!’* (participant 15) *‘At about seven years, I had surgery…so I became female, had a new name, new clothes and a new primary school… I was just told what was going to happen…I suppose I’ve compartmentalised it…’* (participant 12)
Negative affect and cognition	*‘I don’t know if it’s my personality, or if it’s the medication…I’d never be able to differentiate…is it the medication or is it me? Because I’ve never been without medication…I’ve had lots of lows…lots and lots of lows…’* (participant 21) *‘When I was growing up, I was always a bit angry about it…It made me a bit resentful sometimes…I’d think ‘Why can’t I just be normal like you?’... There’s a lady at work and she’s expecting her baby…but I think sometimes it does create a slight jealousy in me that I don’t really like…’* (participant 1)
Low self-esteem and negative self-beliefs	*‘It did make me feel a little bit different…I suppose it did just make me feel a bit different…’* (participant 1) *‘…because of the shame…I was told ‘Never tell anybody, never talk about it'…just keep it to yourself and your family…especially when I was younger, I had this… there was such a desperate sense of shame… It’s* [CAH] *not something that I share with many people…I’m very private and I think because you get used to keeping a secret for so long’* (participant 10) *‘You don’t want people to know you’ve got CAH because they’ll go and google it’.* (participant 6)
Negative body image	*‘ I’ve been very self-conscious about the scarring…the stretch marks on the thighs’* (participant 15) *‘…the skin… the body hair…you’ve got all this hair! On your stomach, on your bum, on your back…and that’s where boys have it, not girls’* (participant 23) *‘I had an enlarged clitoris and I just really struggled with that…that was just really hard, I thought I was a freak...’* (participant 5)
Maladaptive coping strategies	*‘I had lots of binge purge cycles…exercise, bulimia…periods of restriction… if I have to take a lot of medicine for any period of time, it comes back…’* (participant 7) *‘She took a lot of the wrong ones [tablets] and not enough of the other ones, and ended up feeling really, really poorly…but it would take four days for her actually be in a life-threatening condition…’* (participant 14) *‘She has tried to commit suicide…a couple of times… she always says ‘Oh I wish I wasn’t here!’’* (participant 19)

Negative affect and cognition were prevalent in both sexes, with twelve participants reporting anxiety and/or depression. Participants also felt resentment and anger at having the condition, and envy at those unaffected. Although many participants had positive self-esteem, many female participants (*n* = 11) perceived themselves as different, defective, and alone. Feelings of guilt, shame, and secrecy, often instilled in childhood, eroded female self-confidence (Table 3). Women also struggled with their body image owing to excess body hair, weight, genital differences, and skin concerns.

Negative self-beliefs were associated with maladaptive coping strategies, including disordered eating and excessive exercise (Table 3). Some participants reported suicidal ideation and self-harming behaviours, risking adrenal crises through inappropriate medication management and non-adherence. One woman attributed her involvement in abusive relationships to a lack of assertiveness and ‘learnt compliance’ (participant 10) from childhood medical treatment. Consequently, she had misused alcohol and other substances to manage the cumulative trauma of medical and domestic abuse.

### Social functioning

CAH impacted the social functioning of women who reported difficulty disclosing their diagnosis, forming friendships, and having limited friendships (Table 4). Women had difficulty and/or delay forming intimate partner relationships because they needed to trust partners with their diagnosis. In contrast, men were comfortable disclosing their diagnosis, providing reassurance to partners on disease transmission and management (Table 4).

**Table 4:** Summary of key sub-themes relating to the social functioning domain.

Sub-theme	Illustrative quote
Social isolation – difficulty forming friendships	*‘…it was very difficult…to make that connection with other people…but I found that making friendships very, very difficult…’* (participant 3) *‘I…don’t…have…friends per se…I always call people acquaintances…’* (participant 20)
Non-disclosure of diagnosis	*‘Friends…I don’t go around telling everybody at all…I would tend to keep it all to myself…but they do know that I have to take steroids… they don’t know any more than that’.* (participant 16)
Difficulty or delay forming intimate partner relationships	*‘It makes you hold back a little…because you think, if I ever get that intimate with a partner, you’re gonna have to explain… you’re gonna have to tell them at some point…when you were born, you had to have surgery…so you don’t…or I didn’t…go out there very much’.* (participant 6)
Difficulty trusting	*‘I’ve had a few relationships, that have been a bit longer term, and that was mainly down to trusting the person quite a bit…and being able to share…’* (participant 2)
Partner need for reassurance	*‘There were times when friends, girlfriends needed reassurance that it wasn’t contagious’.* (participant 4) *‘I know my previous ex…I know the fact that there was…not question marks…but complications with having kids …and I know that can bring up some question marks…and sort of doubts…’* (participant 11)
Dependency on parents or family	*‘I mean she still lives at home with me… she’s traumatised by all these hospital appointments…she wouldn’t go on her own now, she likes me to go with her…so I think “How’s she going to manage when I’m not here?” I think we should probably be further along on working towards independence… so yeah it concerns me…’* (participant 19)
Parental trauma and negative cognition	*‘She was born just after midnight…and it was when the nurses get into a huddle around the cradle…and you think ‘OK! What’s going on here?’…and they put a white…instead of a blue or pink label on the crib, it was a white one…we pushed down the memories…you know, we didn’t want to have to deal with them…and so I have deliberately chosen not to remember things, and therefore some of these things, I just cannot remember at all…’* (participant 14) *‘So my husband at the time, [name]’s father…he was like “Well, we can’t take her home from the hospital, she’ll have to be adopted!”’* (participant 19)
Positive parental outlook	*‘…She [mother] made sure that the message was clear that I was just the same as everyone else, so that it shouldn’t hold me back in any way…’* (participant 2)
Maternal investment in care	*‘She* [mother] *was quite pushy when we were younger and I think as kids we were always a bit embarrassed about it...I’d always say to mum ‘Mum! Stop asking those questions…it’s embarrassing!’ But she needed to know, in order to help us…’* (participant 1) *‘She* [mother] *was also appalled that I was starting to go to see a consultant without her ’cause she was always in the room… it* [CAH] *was a blessing and a curse, because for her she could wrap me up in cotton wool’. (*participant 17)
Differences in parental treatment of siblings	*‘Mum’s always insisted that he [brother]…that it was worse for him…and it’s very bizarre thing…I was in the hospital so much more than him!..She says to me ‘Oh, but you’re fine with all of that, aren’t you now?’ and I can’t even begin!’* (participant 10)
Societal views around intersex	‘*I have basically come across calls for people with CAH to come and join the trans umbrella…and I find that personally quite offensive…intersex is a massive umbrella of many different things…but I don’t consider myself to be part of…I consider CAH should be part of the umbrella…but it’s a specific thing with specific treatment…’* (participant 7)
Peer support and patient advocacy	*‘…I’ve started going to that CAH group, which is literally the first time I’ve ever met anyone with the illness…but also it had quite a profound effect, meeting everyone six months ago… I feel a bit more settled actually…because it’s nice to meet people just with similar experiences…’* (participant 5)
Support from friends and family	*‘…you know, my sister’s got two girls…so I’ve enjoyed them…’* (participant 12) *‘I think I’m very, very lucky though, that I’ve had a very good support network around me…’* (participant 2)

Complex family (parent–child) dynamics were evident with a few adult CAH daughters reportedly dependent on their mothers (Table 4). Some participants noted parental trauma, shame, and guilt from their child’s diagnosis of a genetic condition. Nevertheless, other participants reported their parents had a positive outlook, empowering them to live a normal and fulfilling life. Mothers were often actively involved in the child’s medical care, while many fathers were considered detached. Some participants viewed maternal involvement in childhood medical care positively, ensuring access to appropriate treatment and supporting transition to independence in adolescence. Others perceived mothers to be overprotective and reluctant to allow their child medical autonomy in young adulthood (Table 4). Two participants noted CAH was a factor in the breakdown of parental relationships, fuelling feelings of guilt in the child.

Several women were uncomfortable with CAH being labelled as intersex because of societal views around gender. They felt strongly that CAH is an endocrine disorder with a specific treatment (Table 4).

Social support was a mediator for HRQoL. Engaging with peer support and patient advocacy was seen as empowering and had a positive impact on participant well-being (Table 4). Peer support reduced feelings of isolation and difference, allowing individuals to share experiences and obtain advice. Participants also reported the support of family and friends and involvement with their children was a source of joy (Table 4).

### Economic or occupational and environmental factors

CAH did not impact participants’ academic ability to work, with all participants of employment age working or studying. Participants noted fulfilling careers, the ability to work nightshifts and unsocial hours. CAH had impacted the career choice of some participants, preventing them from pursuing careers in the military, fire service, and competitive sport (Table 5). Some participants experienced workplace discrimination, resulting in a reluctance to disclose their medical condition to employers (Table 5). Support from colleagues was variable, with some participants reporting supportive co-workers, whereas others perceived colleagues lacked empathy. Despite childhood reports of a reduced motivation to work and school absenteeism due to illness, most participants (*n* = 18) were university-educated.

**Table 5:** Summary of key sub-themes relating to the economic and occupational, environmental, personal beliefs and outlook, and general health perception domains.

Health-related quality-of-life domain	Sub-theme	Illustrative quote
Economic and occupational factors	Impact on career choice	*‘I’ve done night shifts, weekends and everything else…’* (participant 4) *‘I always wanted to join the military and obviously the condition, you can’t do that…’* (participant 11)
	Workplace discrimination and disclosure of diagnosis	*‘It caused me so many issues at work, you would not believe! I basically ended up having to leave a job because of it [CAH]… when I’m applying for jobs it says state when you have a disability…but when I stated it, I wasn’t getting a job, but then when I didn’t state it, I did hear back from the jobs… I would just wait until I was in the job…wait until I could show that I can do my job just like everyone else…and then when I felt comfortable, I’d tell my team… so I told my boss, and he was really angry and said I was selfish and I had put everyone at risk by having this life-threatening condition and not really telling anyone’.* (participant 23)
	Impact on schooling	*‘I wasn’t the best school learner…I think, as a school child, I kind of couldn’t be bothered then’.* (participant 21) *‘…I had a lot of time off school…’* (participant 6)
Environmental factors	Financial resources	*‘I’ve had to self-fund every bit’.* (participant 17)
	Access to healthcare	*‘So at some point, I may well relocate to a hospital that isn’t an hour and half, two hours’.* (participant 22)
	Environmental pollution and opportunities for leisure or exercise	*‘The air is so polluted you’re really breathing it deep into your lungs…so that is a very tricky thing…plus a lot of stomach issues and a very sedentary lifestyle, because of the heat and everything and the humidity …there’s nowhere to go for a walk or a run really’.* (participant 15)
Personal beliefs or outlook	CAH part of identity	*‘…it is integral to who I am…it’s definitely part of me’.* (participant 10)
	Acceptance of the condition	*‘For myself, it’s just the norm …I live my life as well as I can, with my friends and my family and work…and I just do what I can to live a normal life really…and ultimately, I don’t think there’s anything really…as long as I take my medication…It’s just me, this is my life…this is what I am used to’.* (participant 21)
	Positive outlook on life	*‘I feel really positive now…I’m happy! I’m enjoying life!’* (participant 6)
General health perceptions	Fulfilment of life aspirations	*‘It doesn’t really stop me doing what I want…I just got on with it! That’s an age thing, you just become more responsible, don’t you? But it hasn’t stopped me…’* (participant 16) *‘I’m proud of myself, of what I’ve achieved…but you just think, ‘Could I have done so much more?’ but again, we’ll never know…’* (participant 3)
	Perceptions of being sicker than others	*‘I think that because of my condition, when I get unwell, I tend to get very unwell, quite quickly… it’s probably had me in hospital more than the average person…’* (participant 2)
	Avoidance of risks to health and well-being	*‘I can’t take risks anymore because I really want good health and wellbeing…because it doesn’t take a lot to dip down, if I’m honest’.* (participant 10)
	Concerns for future health	*‘I worry about the long-term impact…you know, am I going to make it to my fifties, sixties with decent mobility and decent life…you know, I worry am I going to end up breaking my bones in future because of all the steroids use? I’m worried that I’m going to need a permanent carer….so yeah, I do worry about the future’.* (participant 20) *‘I think about the menopause…I think about preserving my bones…I think about some of that lifestyle stuff’.* (participant 7)
	Expectation of health deterioration	*‘Well…I think that things get worse really, as you get older, don’t they?’* (participant 16)

Access to healthcare and medicines impacted participants’ HRQoL. Participants reported travelling long distances to receive specialist care and restricted access to new medicines (Table 5). The financial cost of private healthcare in the USA and UK was also noted as a burden (Table 5). The living environment was noted to impact health. Participants commented that air pollution, heat, and humidity in some countries contributed to pulmonary issues and limited opportunities for exercise (Table 5).

### Personal beliefs, outlook on life, and general health perceptions

Participants reported that CAH is an implicit part of their identity. Some participants noted acceptance of the condition and a positive outlook on life (Table 5). However, some participants considered that their outlook was influenced by having the ‘mild’ (participant 15) simple virilising CAH, despite having undergone surgery.

Although participants reported living a fulfilling life, two women felt CAH had reduced their life aspirations. Some participants perceived themselves to be sicker than others and were cautious, avoiding risks to health and well-being (Table 5). Women were concerned about future health, particularly the impact of CAH on ageing and life expectancy, menopause, and the long-term effects of glucocorticoids. Thus, a few women expected their health to deteriorate more than normal with age (Table 5).

### Transitions across the life course

Different factors impacted HRQoL across the life course. During childhood, the need for medication and school absenteeism for hospital appointments/treatment set participants apart from peers. Some women, who had undergone genital surgery or experienced precocious puberty, were consciously aware of being physically different to peers. Feelings of shame and secrecy were often inadvertently instilled by parents during childhood, eroding self-esteem. Consequently, forming friendships could be challenging, and some participants were bullied. Participants often reported being over-medicated in childhood, with symptoms of glucocorticoid excess problematic. In adolescence, women struggled with their body image and some were confused about their sexual identity. Sexual debut was often delayed because of anxiety over genital differences, intimacy, and acceptance. Both sexes were concerned about the impact of CAH on fertility and reproductive choices, occasionally adding strain to relationships. As participants aged, concerns about the long-term effects of glucocorticoids became salient.

## Discussion

This study provides an in-depth insight into the wide-reaching impact of CAH on HRQoL. The findings show that CAH is part of an individual’s identity but does not define them. Individuals with CAH can live a satisfying life, achieve academic and career success, and fulfil reproductive goals. However, CAH presents unique challenges across the life course, which can negatively impact HRQoL, particularly for women.

Contrary to previous research (9, 10, 13, 14), participants reported that CAH and associated medical treatments had minimal impact on their physical functioning. However, CAH-specific effects were problematic when glucocorticoid doses were sub-optimal (8, 10). In previous research, impairment of physical functioning was attributed to a lack of energy and vitality (9, 10, 11, 13, 22). Participants reported lethargy as a symptom of adrenal insufficiency. Thus, optimal glucocorticoid replacement, to prevent adrenal crises, normalise androgen levels, and avoid iatrogenic hypercortisolism, is key to ensuring good HRQoL.

Previous HRQoL studies in CAH have shown that the condition is associated with impairment of emotional and mental health, notably higher levels of negative affect and increased prevalence of suicide attempts (8, 9, 10, 11, 12, 13, 14). Similarly, reports of negative affect were common in our participants. Consistent with previous research (6, 33), the psychological well-being of women was compromised by trauma from repeated childhood genital examinations and/or surgery and the associated loss of agency and body ownership. Participants harboured feelings of anger and resentment towards doctors for ‘abusive’ care, with some considering their parents enablers of this treatment. This study supports previous research that women with CAH have impaired body image and negative self-esteem (16, 18). The focus on genital anomalies, the need for ‘corrective’ surgery, excess weight, and androgenic effects are a source of stress and anxiety to women, resulting in some adopting maladaptive coping strategies. The shame and stigma of being different to peers fosters a fear of rejection by family, friends, colleagues, and potential partners.

Consistent with previous research (8, 16, 34), CAH had an impact on family dynamics. Parental trauma, stemming from the child’s diagnosis of CAH and the actions of healthcare professionals, fosters a sense of guilt, shame, and stigma, and parental overprotection was evident. Although supportive in early years, parental control prevented young adults from assuming independent management of their condition. Consequently, some adults with CAH had formed dependent relationships with their parents. Nevertheless, parental psychological support is imperative to facilitate adjustment to the diagnosis and enable them to effectively support their child throughout life.

Women reported impaired social functioning (18). Forming friendships and intimate partner relationships was difficult for women because of low self-esteem, shame, and fear of rejection (6, 18, 34). CAH has a significant negative effect on female sexual well-being and reproductive choices, notably delayed sexual debut, fewer partners, and fewer children (12, 13, 16, 35). Approximately 50% of women report that CAH affects their sex life (16, 35). Women who have undergone genital surgery report issues with sexual function (dyspareunia, stenosis, and altered clitoral sensitivity) (35). This study also highlights that psychosexual impairment is related to defective body image, dissatisfaction with genital appearance and problems with psychosexual identification. Non-heterosexual orientation is more common in women with CAH (16, 35). Although gender incongruence was not reported, this study supports previous research that women’s confidence in their gender identity and self-esteem can be undermined by their perceptions of gender atypical behaviour (6).

In contrast, impaired social functioning was not reported in men with CAH. Previous studies indicate that men with CAH have fewer intimate partner relationships and are significantly less sexually active, with 40% reporting erectile dysfunction (8, 9). While male sexual dysfunction was not apparent in this study, our sample was limited to four young men. Furthermore, it is conceivable that the men may have felt uncomfortable sharing details of their sex life with a female interviewer. However, men did report concerns about TARTs. Indeed, fertility and reproductive choices were a concern to both sexes, placing a strain on relationships (8, 16). Consistent with existing research, some participants had actively decided against parenthood because of the fear of passing CAH to offspring or that parenthood would compromise their health (6). The study highlights the importance of addressing reproductive goals in maintaining good HRQoL and the need to explore reproductive choices with patients.

To our knowledge, this study is the first in-depth qualitative study to reveal the breadth of factors that impact HRQoL of people with CAH living in different countries across their lifespan. However, participants were self-selecting, typically highly educated, and few men participated in the study. Consequently, the results may not be generalisable to the wider population with CAH. Further work is needed to explore HRQoL in a wider demographic population of people with CAH.

This study outlines the multitude of factors impacting HRQoL in CAH that are not captured in existing HRQoL measures. It is evident that CAH has a profound impact on women, whereas the condition affects men to a lesser extent. Further work is needed to define relevant questionnaire items that reflect the domains and acquire evidence of measurement properties. This would require input, particularly from men with CAH. A CAH-specific HRQoL measure, capturing these identified domains, is now needed to support clinicians in delivering effective patient-centred care and improve patient experience and clinical outcomes in this under-appreciated disorder.

## Supplementary materials





## Declaration of interest

KLJ received honoraria from Neurocrine Biosciences. DAR participated in advisory boards and received honoraria from Neurocrine Biosciences. MWOR participated in CAH clinical trials sponsored by Spruce and Lundbeck. NK participated in advisory boards and received honoraria from Neurocrine Biosciences. KLJ, SE, SFA, NK, SL, MWOR, JWT, and DAR are members of the Congenital Adrenal Hyperplasia Adult Study Executive 2 (CaHASE 2) steering committee. The CaHASE2 study is supported by an investigator-initiated trial grant from Neurocrine Biosciences to the Society for Endocrinology. S Faisal Ahmed is Editor-in-Chief of Endocrine Connections. S Faisal Ahmed was not involved in the peer review of this manuscript, on which he is listed as an author.

## Funding

This work was supported by the Living with CAH patient support group.

## Data availability

Data are available from the corresponding author on reasonable request.

## Ethics

Ethical approval was provided by Cardiff University, School of Medicine Research Ethics Committee (ref number: 24/32).
