# Supplementary material for: Factors affecting the quality of life of adults living with congenital adrenal hyperplasia: a qualitative study of lived experience
Source: Endocr Connect. 2026 May 12;15(5):e260033. doi: 10.1530/EC-26-0033 (PMC13188198; doi:10.1530/EC-26-0033)
Supplement: Supplementary file 1 [file supplementary_materials_1.pdf]

Supplementary Information – Disease-specific effects relating to adrenal insufficiency, corticosteroid excess, androgen excess, urogynaecological and testicular symptoms.

Theme	Sub-theme
Adrenal insufficiency	**Adrenal crises:** *"I had a couple of adrenal crises between 16 and 22…I'd start to go into adrenal failures…I'm hospitalised, I think it happened maybe four times in that span…I did my master's at [University]…I had final crisis…I had pneumonia and that turned out into an adrenal crisis…"* (Participant 7)
	**Memory issues/brain fog:** *"…I was complaining about memory issues…There's always been, at the back of my mind, this problem with remembering things…I can't work out what I need to do, or where I need to go to get what I need…"* (Participant 3)
	**Headache:** *"…as I grew up, they introduced a third dose [hydrocortisone] because I was starting to get headaches in the afternoon…"* (Participant 6)
	**Abdominal pain:** *"…If I don't take them [steroids], I get headaches and I start to feel the side-effects…headaches and pains in my stomach and stuff."* (Participant 20)
	**Light-headedness and weakness:** *"I do have, what I say to my girlfriend, "I'm having a hypo!" and she says, "No, you're not!" But I feel really weak…I need to have something…I feel tired, I feel lightheaded…I feel like I am low on sugars, you know. And she's like "No, you're not!" But I do feel like that, you know?"* (Participant 21)
	**Hypoglycaemia:** *"…My medication feels like it's spot on…there were periods before when I'd maybe feel, even though I had taken my tablets, I'd get some of the signs that I would normally get if I hadn't taken my tablets…I'd maybe get some sort of cold sweats, or I'd be feeling like…almost like, I think they're maybe the signs of low blood sugar. Feeling a bit faint, feeling like I'm just craving sugar…and I don't really get that any more, so it feels like it's really well managed."* (Participant 2)
	**Lack of energy:** *"Since I've had that adrenal crisis and I've got older…it definitely feels more like these adrenal lows…not necessarily crisis…but these when I'm feeling like very lethargic…and I know I'll be ok and I don't need to go to hospital…I can't go out and do anything because I basically can't move or talk, because I'm so out of it…it's very debilitating…"* (Participant 23)
Corticosteroid excess	**Weight gain:** *"My body shape changed, I had a lot of bloating in my stomach where I normally don't…I also immediately gained something life five kilos and it was in such as short time that calorically, to work that out, I would have or have been eating something like ten thousand calories a day!"* (Participant 7)
	**Immunosuppression:** *"I also found that being immunosuppressed on the steroids made me ill more frequently…I found that every winter, I was always on antibiotics for something…I had a lot of strep…a lot of ear infections, sinus infections…pneumonia…just all round illness…"* (Participant 7)
	**Bruising:** "Bruising more easily…**"** (Participant 18)
	**Deteriorating eyesight:** *"My sight isn't particularly good…mostly just in one eye, a little bit in the other…again that's long-term steroid use…"* (Participant 16)
	**Hyperglycaemia and diabetes:** *"They then put me on dexamethasone, and I think at that point, around those years, was when the diabetes developed, the cholesterol developed…"* (Participant 3)
	**Insomnia/difficulty resting:** *"I had been feeling like I had been on too much dexamethasone for quite some time…feeling absolutely beside myself, ravenous, dizzy if I tried to eat a normal amount of food…insomnia…"* (Participant 7)
	**Uncontrollable eating:** *"So then she goes on to a tablet form…and suddenly her growth…she's growing enormously…and there was no way to like stop her growing, stop her eating….and I don't think it's all what she eats at home, because everybody else's slim…she always wanted to eat and eat and eat…"* (Participant 19)
	**Gastro oesophageal reflux disease:** *"I have reflux from all the steroids."* (Participant 16)
	**Hallucinations:** *"I understand that but he put me on prednisolone which gave me paranoid hallucinations…"* (Participant 7)
	**Hypercholesterolaemia:** *"My cholesterol is high…but the steroids and the HRT patches and the Prolia [denosumab] all tend to increase cholesterol levels…"* (Participant 21)
	**Hypertension:** *"I can suffer from hypertension if I have too much cortisol or manmade cortisol."* (Participant 17)

Theme	Sub-theme
Corticosteroid excess	**Joint and soft tissue injuries:** *"I was getting injuries…from my late forties onwards, I had pains in my knees from arthritis…I've had problems with my right foot and my lower spine…"* (Participant 15)
	**Mood swings:** *"I think it was partly the medication…I was just up and down…I was just all over the place…I'd get into what I called these negative states, and it fells like…you can't control where you're at! It's such a state of…shouting and upset…and you're just sort of like, you know, really all over the place…and nothing soothes it except trying to rest."* (Participant 10)
	**Moon face:** *"With a moon face, yeah…we did have that ever so slightly when we were about ten or eleven…when I think they overmedicated use ever so slightly…"* (Participant 1)
	**Osteoporosis/osteopenia:** *"Up to the ripe old age of nearly 35! I've been diagnosed with osteopenia…broken this arm twice…my right arm twice…"* (Participant 6)
	**Striae and thin skin:** *"I felt my skin was getting very thin…I was getting a lot of melasma, striae…red marks all over my stomach…"* (Participant 7)
Androgen excess	**Acne:** *"Bad skin…and obviously, you think, my mum was very dark haired, and she was like "Oh, I used to have a lot of body hair…" But this was an excessive amount for a woman I think…and bad skin…My skin had gone really bad again"* (Participant 23)
	**Hirsutism:** *"I also get the excess hair growth very badly because of the imbalance of the testosterone, which I've had for as long as I can remember."* (Participant 20)
	**Deep voice:** *"…You can hear my voice now, how deep it is."* (Participant 3)
	**Absent/irregular periods:** *"I never had periods until I went on the pill when I was 18…they had me on it for maybe three months and then they took me off it and then I basically bled every two weeks…very heavy…"* (Participant 20)
	**Ovarian cysts and polycystic ovaries:** *"Oh yes, aged 35, I was found to have large ovarian cysts, so my ovaries were removed…"* (Participant 12)
	"I was also told I have PCOS and that's something that a lot of women..." (Participant 1)
	**Restricted height:** *"The one thing that has always frustrated me all my life is my height…because I'm only short! I'm five foot three…"* (Participant 21)
Uro-gynaecological symptoms	**Incontinence secondary to surgery:** "Ever since I've had those surgeries, I've had incontinence…I've had lots and lots of investigations…I live with it all the time, so I sort of forget it's a big problem…like I wear incontinence pads daily…" (Participant 20)
	**Vaginal stenosis and scarring:** *"I did have problems initially having intercourse because of the narrowing of my outside area."* (Participant 17)
	"I don't believe my…area down below…is normal…because I have scar tissue that has never gone from when…they messed around with me…" (Participant 20)
	**Urinary tract infections:** *"I get quite a few urine infections and things…"* (Participant 16)
	**Labial/vaginal infections secondary to surgery:** *"…had appendicitis when I was about 13, which was a bit of a giggle…took my appendix out…having an operation triggered my periods which meant "Oh dear! Might need some more surgery here!" because things weren't flowing as they should do, shall we say. So I had a whole load of different infections and nastiness and horrible things around that…"* (Participant 6)
	"Part of the problem of the surgery when I was eleven because when they did the clitoral reduction…they left gaps inside which sounds odd but…these gaps can get debris and you basically end up with an infection inside which is painful." (Participant 9)
Testicular symptoms	**Adrenal rest tumours:** *"There's been examples of testicles being removed because they believed they were cancerous…and actually it was just an adrenal tumour on there, that could have easily been sorted out…"* (Participant 22)
